# Bacterial immunotherapy: is it a weapon in our arsenal in the fight against cancer?

**DOI:** 10.3389/fimmu.2023.1277677

**Published:** 2023-11-27

**Authors:** Shubhra Sharma, Himani Sharma, Himanshu Gogoi

**Affiliations:** ^1^Amity Institute of Microbial Technology, Amity University Rajasthan, Jaipur, India; ^2^Translational Health Science and Technology Institute, National Capital Region (NCR) Biotech Science Cluster, Faridabad, India

**Keywords:** bacteria, cancer, immunotherapy, tumor-microenvironment, vaccine

## Abstract

Advances in understanding the genetic basis of cancer have driven alternative treatment approaches. Recent findings have demonstrated the potential of bacteria and it’s components to serve as robust theranostic agents for cancer eradication. Compared to traditional cancer therapies like surgery, chemotherapy, radiotherapy, bacteria mediated tumor therapy has exhibited superior cancer suppressing property which is attributed a lot to it’s tumor proliferating and accumulating characteristics. Genetically modified bacteria has reduced inherent toxicity and enhanced specificity towards tumor microenvironment. This anti- tumor activity of bacteria is attributed to its toxins and other active components from the cell membrane, cell wall and spores. Furthermore, bacterial genes can be regulated to express and deliver cytokines, antibodies and cancer therapeutics. Although there is less clinical data available, the pre- clinical research clearly indicates the feasibility and potential of bacteria- mediated cancer therapy.

## Introduction

Cancer brings about psychological and physical anguish to the patient and family. Cancer related deaths are one of the leading cause of demise globally with cardiovascular related deaths at the forefront (1–3). Colon, lung, liver, pancreas, stomach, breast and bowel continue to be the most common organs impacted by the disease (3, 4). Tumorous condition begins with uncontrolled growth of cells and formation of a mass within an organ or tissue. It can be either benign, and grow but do not spread to distant tissues or it can be malignant and spread to other tissues of the individual. Uncontrolled and invasive growth of self- cells beyond the tissue of origination and spread to otherwise healthy tissues leads to metastatic phase of cancer. Genetic and epigenetic alterations resulting from ageing, prolonged exposure to different mutagens, adverse epigenetic factors, and chronic infections results in an increased proliferation, affecting cell cycle through gene functioning in proto- onco and tumor suppressor genes (6–9). Advancements in the field of cancer therapy and diagnostics have led to the development of surgery, chemotherapy, and radiotherapy resulting in increased survival rates of cancer patient world- wide (10). However, due to a diversity of anatomic location of the cancer cells, histologic origin, intertumoral heterogeneity due to genomic alterations and diverse immunological characteristics within the tumor micro- environment have shown the need for more advanced cancer prognosis and therapeutics (11). These inherent properties of tumor cells along with non- specific toxicity of cancer therapy, surgically unremovable and probable drug resistance have prompted the need for development of alternative approaches. Targeted therapy and immunotherapy are two of the promising strategies in the fight against cancer. Targeted therapy aims at abnormalities associated with cancerous cells to increase tumor specificity and reduce toxicity in healthy tissues. Solid tumors are characterized by poor oxygen, low pH, elevated interstitial fluid which supports tumor proliferation, immunosuppression and resistance against conventional tumor therapy (12, 13). Targeted therapy aims to target these abnormalities to increase specificity of anti- cancer therapeutics and reduce non- specific cytotoxicity. Immunotherapy harnesses a host’s immune system to eliminate tumor by activating the immune cells and interacting with the tumor antigens. Recent years have seen profound development with immune checkpoint blockade therapy (monoclonal antibodies against programmed death protein 1; PD1 and cytotoxic T-lymphocyte associated protein 4; CTLA4) (14–17) and chimeric antigen receptor T cell (CAR- T) therapy proving themselves to be powerful anti- cancer therapeutics (18, 19). The sensitivity and efficacy of these therapies however depend on the innate tumor micro- environment and specific ligands expressed by the tumor cells which requires developing personalized treatment strategies. Major efforts are being made to develop alternative approaches that can circumvent the obstacles of the current cancer treatment regimen. Application of therapeutic bacteria as anti- cancer therapeutic has fascinated researchers globally. The application of bacteria and bacterial toxins as anti- cancer agents is more than a century old with initial findings of German physician Wilhelm Busch in 1868, where he observed tumor regression in cancer patients after accidental infection by *Streptococcus pyogenes* (20). His findings were reproduced by Friedrich Fehleisen in 1882 who identified *Streptococcus pyogenes* as the causative agent of erysipelas and shrinkage of malignancy (20). However, the noteworthy reports of bacteria mediated tumor therapy gained momentum after William Coley’s well documented report of bacteria mediated regression of inoperable skin cancer. In late 1800’s, his trials utilizing inactivated bacterial species *Streptococcus pyogenes* and *Serratia marcescens* could successfully treat patients with carcinoma, lymphoma, melanoma and myeloma (21, 22). The contribution of William Bradley Coley in the field of cancer immunotherapy using bacterial toxins made researchers realize the potential of the bacteria in tumor treatment (23). Furthermore, pediatric vaccinations have been correlated with decreased incidences of childhood cancer. By utilizing population data and meta- analysis, various research groups have attributed that exposure to bacterial or viral vaccines during infancy serves as immunomodulators leading to a reduction in cancer incidences (24–30). These reports further strengthen the potential of bacteria and bacterial mediated therapeutics to treat cancer.

**Figure 1:**
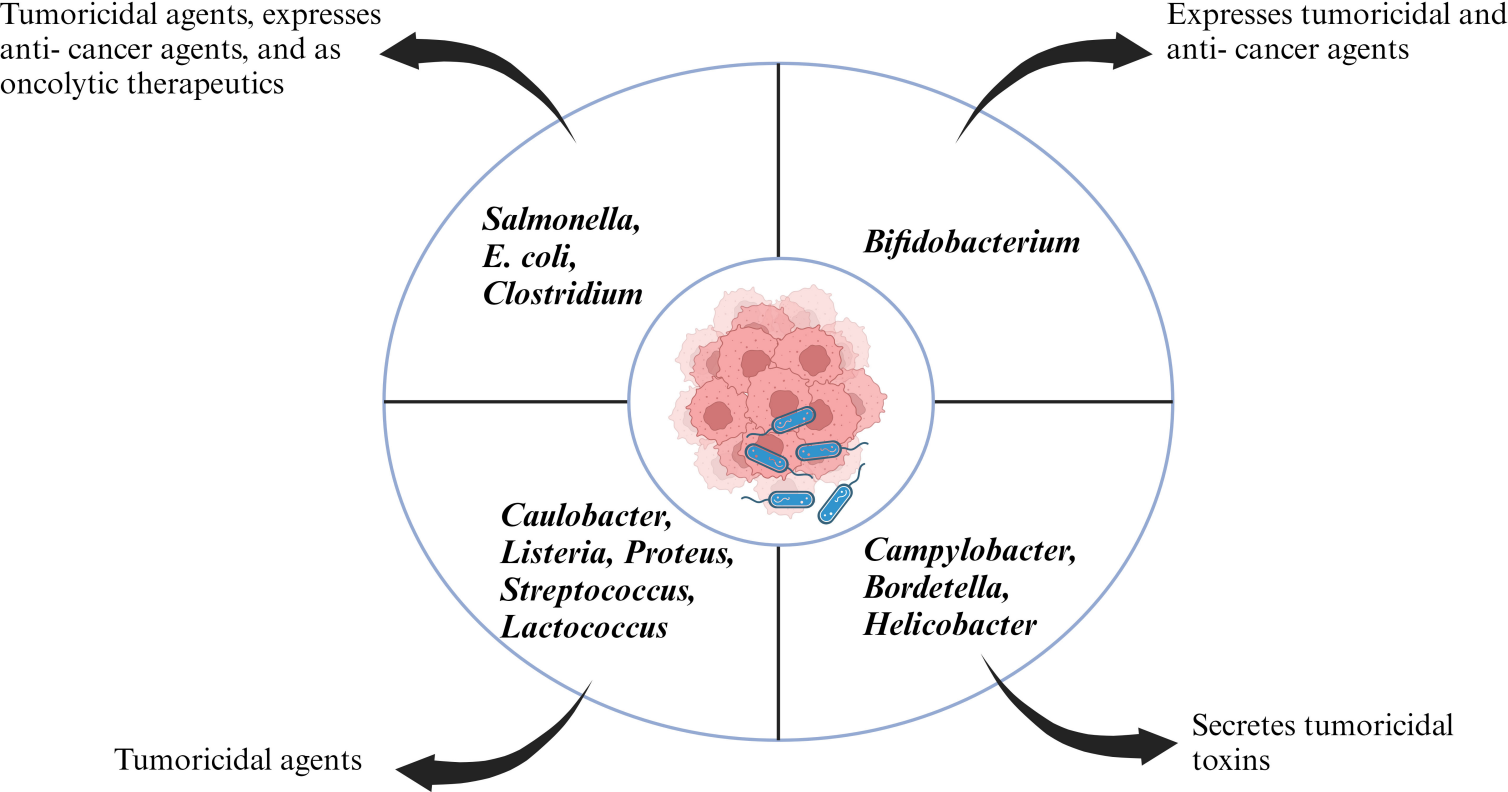
Representative figure of different bacterial species acting as anti-tumor therapeutics.

Coley’s revolutionary work opened the doors for new experimental candidates with bacterial species ranging from *Salmonella*, *Clostridium*, *Listeria*, *Lactobacillus*, *Escherichia*, *Pseudomonas* and *Caulobacter* with some bacterial species going to clinical trials (**Figure 1**). The initial application of bacteria mediated cancer therapy was met with criticism due to the adverse effects ranging from bacteria mediated septic shock and death due to immune- suppression. However, genetic engineering enabled researchers to manipulate the microorganism and knockout/knockdown genes which contributed to reduction of cytotoxic proteins, or virulence factors but enhanced penetration of tumor microenvironment and activate the host’s antitumor immune system (31–33). Bacilli Calmette- Guerin (BCG) has been a success story of bacteria mediated cancer immunotherapy and approved by FDA for treatment of superficial bladder cancer (34). Bacterial cell wall components like lipopolysaccharide and peptidoglycan can act as pathogen associated molecular patterns (PAMPs) and potentiate the host immune system. Intratumoral delivery of these bacterial components can mimic an *in- situ* vaccine and activate antigen presenting cell’s (APC’s) like dendritic cells which can phagocytose tumor antigen’s and migrate to the draining lymph nodes where they can prime cytotoxic T cells (35). In an article by Chowdhury et al., the researchers have reported quorum sensing genetically modified bacteria encoding for a single- chain antibody fragment targeting the phagocyte inhibitory ligand CD47 in macrophages. This strategy led to development of a systemic anti- tumor immunity resulting in tumor regression, metastasis prevention and prolonging survival of mice (36). Chemotactic mediated bacterial targeting and accumulation within tumor’s has been associated with tumor hypoxic micro- environment, pre- dominant expression of clusterin, serglycin, TGF- β2 etc (37). Furthermore, research groups have reported bacteria as a vector to deliver anti- cancer therapeutics (38–40). Various mechanism hypothesizing the role of bacterial enzymes like bacteriocin, bacterial biofilms and activation of host inflammasome pathway in antitumor immunity have been reported (41–43).

In this article, we have summarized the recent progress in the field of bacterial mediated cancer immunotherapy and it’s future as one of the potential weapons in the arsenal in the fight against cancer. We also discussed the mechanism associated with tumor microenvironement mediated egression of bacteria and the challenges associated with this strategy.

## Cancer progression, the human body, and therapeutics

The ability of the human immune system to distinguish between self and non-self enables it to defend the body against both foreign and endogenous illnesses. The human immune system is a complex network comprising of white blood cells as well as lymphatic organs such as thymus, spleen, tonsils, and lymph. The immune system recognizes multiple dangers and removes them to maintain homeostasis in the nodes, lymphatic vessels, and blood. Cancer is a “developmental disorder” which arises when cells divide uncontrollably and spread into surrounding or distant tissues. Host immune system acts as a double- edged sword in cancer metastasis. CD4^+^ Th1 cells, CD8^+^ T cells, NK cells, IFN-γ, M1 macrophages uninterruptedly function to be anti- tumor, while regulatory T cells (Treg), M2 macrophages, Th17 cells, overexpression of TGF- β, and IL- 10 facilitates immune evasion of the tumor cells (5, 44). Understanding the relationship between cancer cells and the immune system is necessary to comprehend how immunotherapy has developed into a mainstay of cancer treatment. While there are many distinct types of immunotherapies with diverse mechanisms of action, they primarily rely on the host immune system to destroy tumor cells, unlike chemotherapy, which kills cancer through cytotoxic qualities (45).

Cancer development and progression can be initiated by genetic and functional abnormalities. Epigenetic modifications such as DNA methylation, histone acetylation, and genetic mutations can lead to an altered gene expression and abnormal cell functionality (46–48). Cancer and its treatment negatively affect the physiology the patient, such as the removal of body parts, colostomies, hair loss, and other things. Several of the modifications are apparent to others, like hair loss, while others, like colostomy, are less obvious. For a cancer survivor, the physical changes brought on by the disease or treatment are frequently profound. Psychosocial cancer research has long recognized the significance of a modified physical appearance following treatment when patients evaluate their quality of life (49).

The most common cause and hallmark of cancer-related mortality is metastasis, and more than 90% of cancer related death occur due to metastasis. “Metastasis” refers to the growth of secondary tumors in an area of the body that is remote from the main site of tumor growth. Understanding the molecular mechanisms underlying the metastatic process needs to be established in order to identify therapeutic windows for effective interventions. Some of the molecular underpinnings of this dispersion process have been revealed as a result of the ongoing evolution of cancer biology research and the introduction of new paradigms in the study of metastasis which are influenced by genetic and epigenetic changes inside the host’s tumor cell and its surroundings. Large quantities of cancer cells are discharged into the bloodstream every day in cancer patients, although melanoma research in animal models suggests that only 0.1% of tumor cells spread to cause metastasis. Targeting metastatic seeding and colonization remains a difficulty despite substantial studies. The notion that metastatic cancer cells dynamically and selectively change their metabolism at every stage of the metastatic cascade has just recently come to light. In addition, many metastases differ metabolically from the primary tumors from which they arise, allowing for survival and expansion in the new environment. Knowing the kinetics of this process as well as targeting new metabolic qualities that emerge in metastases may enable their elimination or offer molecular therapeutics that may slow or even stop the spread of cancer (50, 51).

Early efforts to treat cancer by Paul Ehrlich in 1900’s using aniline dyes and alkylating agents were not very encouraging (as reviewed by DeVita and Chu in (52)). Research by Yale pharmacologists, Alfred Gilman and Louis Goodman on mice bearing a lymphoid tumor demonstrated a marked regression of the tumor when it was treated with a compound, nitrogen mustard (53). The same regression was observed when a patient with non- Hodgkin’s lymphoma was treated with the compound (53). These results brought a ray of hope to cancer treatment in the mid 1940’s (54, 55). However, remissions turned out to be brief and incomplete which created an air of pessimism in that period (56). However, discovery of fluoropyrimidine 5- fluorouracil (5- FU) by Charles Heidelberger at the University of Wisconsin for treatment of nonhematologic cancers can be stated as a pathbreaker in chemotherapy. 5- FU still remains the cornerstone for treatment of colorectal cancer. 1960’s and 1970’s witnessed the advent of combination chemotherapy with a combination of 5- FU, cyclophosphamide and methotrexate against metastatic cancer which demonstrated promising results (57). Despite enhancing survival, anti- cancer chemotherapy still remains a concern for both patients and clinicians. Chemotherapy induced nausea and vomiting (CINV) are amongst the most common side- effects with patients undergoing chemotherapy. Other severe side effects include oral and gastrointestinal mucositis leading to anorexia, weight loss, anemia, and fatigue. Hypersensitivity, cardiovascular toxicity and nephrotoxicity have been reported to be associated with platinum chemotherapy. Chemotherapy induced peripheral neuropathy (CIPN) like depression, ataxia, insomnia are other side effects related to anti- cancer drugs like platinum- based, vinca alkaloids, taxanes, and proteosome and angiogenesis inhibitors (58). Hence, new approaches to improve tolerance and reduce sequlae of chemotherapy is an urgent need of the hour (59).

## Cancer and bacterial relationship within the host

The normal microflora present on the human body consists of different bacteria, protozoa, fungi and viruses. This microflora interacts with each other and helps in regulating the human metabolism and immunity. Recent research on the gut and resident microflora have been found to affect the host’s antitumor immunity (60). The gut microbiota is frequently cited as one of the most crucial elements in maintaining a healthy homeostasis. In some studies, probiotic bacteria have been demonstrated to exhibit antitumor activities and to play a substantial role in immunomodulation. Short-chain fatty acids, which influence cell death and proliferation are known signaling molecules in the immune system, can be produced by bacteria and can lead to the detection and destruction of potential carcinogens. Due to their influence on immunomodulation, lactic acid bacteria found in the gut have been shown to play a part in the reversal of carcinogenesis. This finding supports the notion that bacterial metabolites interact with immunological and epithelial cells (61). Furthermore, probiotic bacteria can influence the production of anti-inflammatory cytokines, which are crucial in the elimination of tumor cells. Probiotic bacteria can also influence and activate phagocytes to get rid of cancerous cells. Synergistic application of radiation and probiotic bacteria have improved the immune system’s ability to recognize cancer cells. The immunity of mice to carcinogens is directly correlated to the presence or absence of an active microbiome. The idea of employing probiotic bacteria as medication delivery vehicles has recently gained traction as a result of multiple articles revealing promising outcomes. Probiotic bacteria and gut microbiota are likely to play a significant role in cancer prevention and therapy over the next several years (61).

Human tumors are known to act as host for bacteria; however, it is unknown whether these bacteria exhibit a commensal or symbiotic relation with the tumors. Bacterial makeup varies with the type of tumor. For instance, bacteria that make monothiol, which can detoxify reactive oxygen species, were more prevalent in breast cancer subtypes that were characterized by elevated oxidative stress (62). In some instances, bacteria can promote tumor development in specific organs. Until recently tumors were supposed to be sterile, but Fu et al., demonstrated that bacteria can colonize tumor and promotes host cell survival to circulating tumor cells by reorganizing actin cytoskeleton and enhancing resistance (63). Moreover modern genotyping techniques involving immunohistochemistry, and fluorescence *in- situ* hybridization (FISH) and 16S ribosomal RNA sequencing have revealed distinct microbiota across 1526 tumors across seven cancer types including breast, lung, ovary, pancreas, melanoma, bone and brain cancer (62).

## Mechanism of action of bacteria as anti- cancer therapeutic

**Figure 2:**
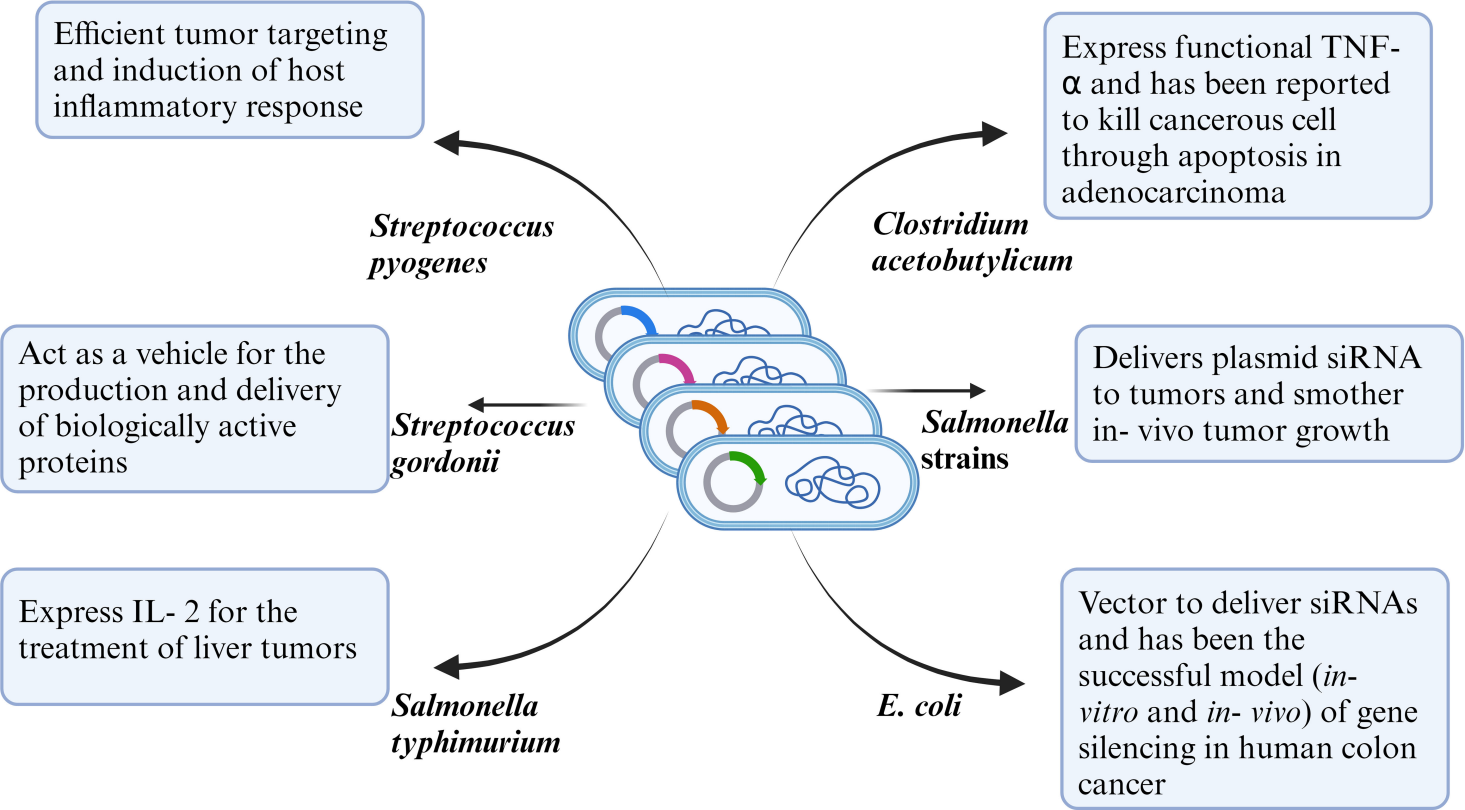
Representative figure of mechanism of anti-tumor efficacy of WT or genetically modified bacterial strains.

Cancer is considered one of the deadliest diseases and a definitive cure for this disease is still the need of the hour. The disease involves proliferation of abnormal cells that invade normal tissues and organs. Along with the traditional cancer treatment approaches like surgery, radiation and chemotherapy, monoclonal antibody-based immunotherapy has been recognized as a milestone in the treatment of cancer. Monoclonal antibodies target tumor cell receptors and induces long lasting antitumor responses (64) . The very first attempt of involving the host immune system for cancer treatment was done by William B. Coley is recognized as the father of immunotherapy (65). Traditional treatment strategies like chemotherapy and radiotherapy resulted in unwanted cytotoxicity, poor drug adsorption, ineffective tumor clearance and developing resistance against anti- cancer drugs. Bacterial immunotherapy has been shown to help in overcoming the problems faced during conventional treatment method. Bacteria and archaea contain numerous bioactive compounds which effectively work against the tumor by inhibiting the growth of cancer cells (66, 67). Injecting tumor targeting bacteria enables the bacteria to colonize the tumor and orchestrate a plethora of immune which results in destructing the tumor cells. Bacteria has been used alone or in combination with conventional methods including radiotherapy, chemotherapy and have shown effective results in inhibiting cancer cell metastasis as well as reduction of tumor. Bacterial species like *Salmonella, Clostridium, Bifidobacterium, Lactobacillus, Escherichia, Pseudomonas, Caulobacter, Listeria, Proteus*, and *Streptococcus* have been explored as potential anti- cancer agents by various research groups (68). Bacterial mediated regression of tumor can be attributed to various mechanisms, likely bacterial species, their intrinsic properties like toxin secretion or pathogen associated molecular pattern (PAMP), genetic modification, delivery vector, synergism with host immune system etc (**Figure 2**).

*Toxin mediated anti- tumor efficacy:* Coley’s toxins consisted of exotoxins secreted by *Streptococcus pyogenes* and *Seratia marcescens.* Additionally, *S. pyogenes* also secretes some pyrogenic exotoxins such as SpeA, SpeB and SpeC. These pyrogenic toxins stimulate CD4^+^ lymphocytes resulting in a rapid release of cytokines (69). Prodigiosin, a low molecular toxin produced by *Serratia marcescens*, *Serratia plymuthica*, *Hahella chejuensis*, *Pseudomonas magnesiorubra* and *Vibrio psychroerythreus* has demonstrated antitumor activity in drug resistant cancer cells like A2780RCIS (MRP1,2 overexpressing human epithelial ovarian cancer cell line), EPG85- 257RNOV (BCRP overexpressing human gastric carcinoma cell line), EPG85- 257RDB (MDR1 overexpressing human gastric carcinoma cell line) (70). On the other hand, *Pseudomonas* spp secretes exotoxins which binds to tumor cell receptors and induces cell death (71). *Clostridium perfringens* produce enterotoxins, an anticancer agent which works by binding to receptors (CLDN3 and CLDN4) highly expressed on colon carcinoma and epithelial tumors resulting in a multi- protein membrane pore complex thereby causing lysis of tumor cells (72). Similarly *Pseudomonas aeruginosa* produce exotoxin T which reduces the growth of B16 melanoma (67). Modified forms of toxins have often resulted in superior efficacy as compared to its natural counterpart. Diphtheria toxin produced by *Corynebacterium diphtheria* is not efficient in cancer therapeutics, but it’s modified form DT modified with the amino terminal (AT) fragment of urokinase- type plasminogen activator (DTAT) has been tested on cell lines and murine model and reported to target vascular endothelial of the tumor, reduced tumor size (73) and resulted in significant regression of human U118MG tumor induced in nude mice (74, 75).

*Enzymatic action:* Different bacterial enzymes have also been reported to act as anti- cancer therapeutic agents. For instance, L- asparginase isolated from *E. coli* have been reported to activate aspargine hydrolysis which resulted in cell death of different tumor cell lines like MCF- 7, HepG2 and SK-LU-1. Moreover, asparginase also acted as anti- neoplastic drug in lymphoblastic leukemia (76). Arginine deaminase isolated from *Streptococcus pyogenes* reduced proliferation of arginine deficient tumor glioblastoma multiforme (77). Likewise, purified pyocin S2, secreted by *Pseudomonas aeruginosa* has been demonstrated to exhibit cytotoxic effects on Im9 (a human immunoglobulin- secreting cell line derived from multiple myeloma) and no effect on normal human cells (78). Microcin E492 secreted by *Klebsiella pneumoniae*, pediocin from *Pediococcus acidilactici* K_2a2- 3,_ nisin from *Lactobacillus lactis* have been reported to exert cytotoxic effects in human malignant cell lines like Jurkat (T cell derived from acute T cell leukemia), RJ2.25 (variant of Burkitt’s lymphoma), HT29 (human colon adenocarcinoma), MCF-7 (human breast adenocarcinoma), HepG2 and head and neck squamous cell carcinoma, but non- toxic on normal human cells (79–83).

*Tumor colonization and regression*: Facultative anaerobic bacterial species also exhibit the property to colonize tumor and replicate within the hypoxic tumor which results in the inhibition of cancerous growth (84). *Salmonella typhi* a flagellated, Gram-negative bacilli is very well known to cause typhoid fever and other food borne diseases. Although pathogenic, it also works as a promising cancer therapeutic agent because of its facultatively anaerobic nature i.e. it can survive in both anaerobic as well as aerobic environment and can occupy both the hypoxic as well as non-hypoxic areas of the tumor (40, 85). *Salmonella* directly infects the tumor cell and induce caspase 1 by inflammasomes and destroy them by apoptosis or regulating autophagy via AKT/mTOR pathway (23).*Salmonella* also mediates T cell and innate immune cell infiltration to tumor site which enhanced killing of *Salmonella* localized tumor (86). Study on *Listeria* spp. infection generates reactive oxygen species by activating nicotinamide adenine dinucleotide phosphate (NADP+) oxidase or elevating intracellular Ca^2+^ levels (87). These ROS induces the immunogenic death of tumor cells and activate CD8^+^ T cells (88).

*PAMP’s and their anti- tumor potential*: Bacterial cell wall consists of pathogen-associated molecular patterns (PAMPs) which have the ability to trigger the immune cells even in the tumor immunosuppressive microenvironment which enhances specific immune recognition and help in the elimination of tumor cells (23). Along with bacteria-based cancer therapy, pathogen-associated molecular patterns (PAMPs) of bacterial membranes has also attracted the world’s attention in generating anti- tumor response. PAMP’s are acknowledged by the antigen presenting cells leading to activation of the T cell mediated immunity. Along with the activation of T cell mediated immunity, toll-like receptors (TLR’s) are also activated, and they are responsible for the production of cytokines like IL-12 and other molecules like CD40. Furthermore, a strong immune response is generated against the cancer cells by the Th-1 dependent immune response and CD8^+^ T effector cells (73).

*Cell wall components as vectors:* Outer membrane vesicle (OMV) of bacteria has also attracted researchers as potential drug delivery vehicle. Outer membrane vesicles from Gram positive and Gram negative bacteria have been coated on polymeric micelles that contained drugs to create an inventive nanomedicine for efficient cancer immunotherapy and metastasis prevention using bioengineering technique (23, 89). The loaded drug within polymeric micelles can exert both chemotherapeutic and immunomodulatory activities to sensitize cancer cells to cytotoxic T lymphocytes (CTLs) and to directly kill cancer cells, whereas OMVs might trigger the host immune response for cancer immunotherapy. Systemic injection of such a bioinspired immunotherapeutic drug would not only greatly limit tumor growth *in vivo* and increase the survival rate of melanoma mice but would also give excellent protective immunity against the development of melanoma. In addition, nanomedicine could successfully prevent lung metastasis of the tumor. The bacterial-based cancer immunotherapy formulation is repurposed by the bioinspired immunomodulatory nanomedicine, which also provides a helpful bioengineering technique to enhance the current cancer immunotherapeutic drugs and delivery systems (89). An alternative approach in cancer treatment apart from OMV’s are bacterial ghosts (BGs) and archaeosomes which have been used as a nano formulation as drug delivery system to deliver anti- cancer therapeutics. Combination of different drugs are incorporated into these biological nanocarriers which are present inside the bacteria and used to kill the cancerous cells (67). These biological nanocarriers work as an efficient drug delivery system because of their certain properties as they are biodegradable, they fuse with the target cells, circulate longer in the body by escaping the immune system and enhance the cellular uptake (67). Implication of doxorubicin loaded OMV from attenuated *Klebsiella pneumoniae* prevented A549 tumor growth in BALB/c nude mice. The formulation was well tolerable and OMV resulted in recruitment of macrophages in the TME (90). In yet another approach, Kim et al., designed *E. coli* protoplast derived vesicles and epidermal growth factor (EGF) tagged protoplast derived vesicles encapsulating doxorubicin/idarubicin. Tagging of EGF on these vesicles resulted in higher accumulation of the drug within the tumor sites and resulted in significant decrease in tumor size as compared to free drug (91). OMV’s stimulate anti- tumor response by secreting cytokines and chemokines like IFN-γ, IL-12p40, CXCL10, TNF- α, IL-6 and have been predicted to be self- sufficient for less destructive tumors like CT26 or MC38 adenocarcinoma (92).

*Genetic engineering of bacteria*: With the advent of recombinant DNA technology, researchers have designed genetically modified bacteria with inherent ability to colonize the tumor. These bacterial therapies not only increase the efficacy and efficiency but also minimizes the toxic effects which normally occur during cancer treatments. A major limitation in developing an efficient cancer therapeutics is overcoming the immunosuppressive tumor microenvironment (TME) which consists of M2 type macrophages, myeloid derived suppressor cells etc (93–95). This TME can be effectively utilized to deliver genetically modified bacteria to be protected from the host’s immune system and exhibit its anti- cancer efficacy. The tumor apoptosis is initiated by the bacteria due to the different interactions that take place between the cancer cells, bacteria, cytokines and chemokines (66). Manipulation of *S. typhi* to express either the proapoptotic Fas ligand or CCL21(chemokine with anti- tumor properties) as a delivery vehicle carrying anti- cancer therapeutics has demonstrated primary tumor inhibition (23). In another study, Sedighi and group utilized and demonstrated *Clostridia, Bifidiobacteria*, and *Salmonellae* species as vectors to deliver suicide genes, tumor associated antigens or for expressing tumor suppressor genes. *Salmonella typhimurium* and *Clostridium butyricum* have been utilized for their inherent capability to selectively colonize the tumor and are used as delivery vectors in mouse tumor models and do not exert any adverse side effects. Sedighi et al. have demonstrated the ability of genetically modified bacteria as opposed to normal bacteria to multiply significantly more in tumorous cells (68). Furthermore, they have also reported rapid tumor regression & regression of cancer has been observed by the help of immunotherapeutic agents such as *Streptococcus pyogenes* infecting the patient with erysipelas (68). Certain bacteria like *Staphylococcus epidermis* colonize the skin and can induce a highly specific systemic immune response. Utilizing this concept, in a most recent research, Chen et al., engineered *S. epidermis* to express melanoma tumor antigens. Skin colonization of the bacteria led to development of tumor specific T cells which were circulatory, could infiltrate local and metastatic lesions and induced tumor regression (96).

With the help of genetic engineering, synthetic biology is ushering in a new age in medicine. The development of designed systems that can intelligently perceive and react to various environments is made possible by this innovative method, which eventually adds specificity and efficacy that go beyond the capabilities of molecular-based treatments. Chowdhury and group designed a non-pathogenic strain of *Escherichia coli* to specifically lyse within the tumor microenvironment and release an encoded nanobody antagonist of CD47 (CD47nb), an anti-phagocytic receptor that is frequently overexpressed in a number of human cancer types (36). In a syngeneic tumor model in mice, CD47nb delivery by tumor-colonizing bacteria boosted activation of tumor-infiltrating T cells, drove rapid tumor regression, inhibited metastasis, and promoted long-term survival. Furthermore, local injection of CD47nb-expressing bacteria triggered systemic tumor-antigen-specific immune responses that inhibited the growth of untreated tumors, demonstrating the feasibility of an abscopal effect brought on by an engineered bacterial immunotherapy. Hence, a safe and local administration of immunotherapeutic payloads that induce systemic antitumor immunity may be accomplished using modified microorganisms (36). In another approach, *Salmonella typhimurium* was engineered to deliver shRNA- expressing vectors targeting *Bcl2* or indolamine 2,3 – dioxygenase 1 (IDO1) significantly silenced the gene in a murine melanoma model, enhanced tumor cell death and prolonged survival (97, 98).

*Exploiting trained immunity:* Along with injecting patients with bacteria which has the ability to express tumor antigens, another alternative way to activate the immune system by utilizing immunodominant T cell antigens from pathogens such as tetanus toxoid, poliovirus or measles. Childhood vaccination against these pathogens generate memory cells which can be utilized for the destruction of tumor cells. This process involves the presentation of immunodominant T cell antigens on the surface of tumor cell infected by the tumor targeting bacteria carrying the expression cassettes for these antigens (99).

## Limitations of bacteria mediated cancer therapy

Although bacteria- mediated cancer therapy has managed to garner attention of the scientific community and have demonstrated its efficacy in *in- vitro* and *in- vivo* models, there are still some unsolved limitations related to potential host immune responses, efficacy and accuracy of targeted delivery and impeded self- reproduction. Innate bacterial toxicity leading to sepsis, chronic inflammation, lymphatic proliferation, potential of DNA mutations thereby resulting in loss of functionality or exaggerated infection, induction of hormones upregulating tumor cell proliferation, production of carcinogenic metabolites which interrupts regulation of cell growth or directly effecting oncogenesis are major concerns associated with the therapy (33, 100, 101). Another major problem reported by Patyar et al., is the incomplete lysis of tumor thereby necessiting the combination therapy with chemotherapeutic treatments (33). This therapy also lacks relevant clinical trials and a reproducibility issue among patients has been a matter of concern. Even the attenuated *Mycobacterium bovis* (Bacilli Calmette- Guerin), the only clinically approved anti- cancer bacterial therapy developed tissue sepsis and high rates of tumor relapse (102).

## Discussion

Although methods such as chemotherapy and radiotherapy are considered the cornerstone of cancer treatment, their results are associated with severe weakness in cancer patients. Moreover, pathophysiology of solid tumors imposes barriers which prevent penetration and efficacy of anti- tumor chemotherapy drugs. With advances in medical technology, conventional cancer treatment strategies have undergone significant improvement. Newer therapies like checkpoint blockade inhibitors and CAR- T therapy have revolutionized anti- cancer treatments. Bacteria can be considered as a promising therapeutic to treat cancer. Their intrinsic properties like hypoxia tropism, self- propelled motility, ability to genetically insert dene or drug make them excellent candidates. Genetically engineered bacteria with reduced virulence but retained tumor targeting have been developed. Several bacteria ranging from *Salmonella* sp, *Streptococcus* sp, *E. coli*, *Clostridium* sp have been described. Intravenous administration of members of *Clostridium* sp*, C. sporongenes*, *C. novyi*- NT, *C. acetobutylicum*, and *C. beijerinckii* have shown to result in tumor colonization and extensive oncolysis. Attenuated *Salmonella* have demonstrated high penetrability into solid tumors which are otherwise unreachable by conventional therapeutic strategies (103). Genetically attenuated *Salmonella typhimurium*, obtained by chromosomal deletion of *purI* and *msbB* genes, resulted in low pathogenicity, strong tumor accumulation and anti- tumor activity. This double mutant strain, VNP20009 have demonstrated its anti- tumor efficacy in different tumor models like B16- F10 murine melanoma, LOX human melanoma, DLD-1 human colon carcinoma (104). In another instance, *Salmonella* mutant strain ΔppGpp enhanced production of TNF- α and IL-1β by macrophages and dendritic cells within tumor cells and induced tumor cell apoptosis (43, 105). Genetically engineered *S. typhimurium* and *C. novyi*- NT have been tested in clinical trials and activated a plethora of host cytokines and chemokines like IL-2, IL- 18, CCL- 21 (106–108). *S. Typhimurium* VNP20009 entered the human clinical trials in 1999 as the 1^st^ bacteria mediated cancer immunotherapeutic agent (109). The efficacy of the strain was poor as it failed to reduce tumor size in 24 patients with metastatic melanoma and in 1 patient with metastatic renal carcinoma which was attributed to a difference in tumor structure and growth rates that effects bacterial penetration, proliferation and clearance within tumors (109). Another factor which was attributed was TLR4- mediated signaling which could be important for tumor colonization and anti- tumor efficacy. *S. typhimurium* strain lacked efficient recognition by TLR4 and failed to colonize tumors sufficiently to suppress tumor growth (110). Clinical trials based on *C. novyi*- NT spores demonstrated extensive tumor destruction when immunized via either intravenous or intratumoral route. However, the strain failed to eradicate all the tumorous cells resulti.ng in tumor relapse (109, 111, 112). Currently, a combinatorial therapeutic based on *C. novyi*- NT strain alongwith anti- PD1 antibody is in phase 1b clinical trials (111) (**Table 1**). Lastly, genetic modifications of bacterial strains can enhance bacteria to respond to stimuli like pH, ultrasound, chemical and thermal which can increase its accumulation at the tumor site. For instance, Qin et al., placed *cytolysin A* under the control of the acid sensitive promoter adiA resulting in the release of ClyA and inhibiting CT26 tumor progression and metastasis (113). Ahmedi et al., demonstrated that utilizing temperature- dependent transcriptional repressors, TlpA39 and Tcl42 in the clinically approved *E. coli* Nissle 1917 (EcN) could lead to a temperature regulated release of anti CTLA-4 and anti PD-L1 antibodies resulting in A20 tumor retardation in a murine model (114). Tumor cells rapidly utilizes glucose via glycolysis resulting in glucose depletion (115). Panteli et al., demonstrated a glucose concentration dependent fusion protein Trz1 transformed in *E. coli* to sense glucose concentration and induce expression of GFP which was under the osmoporin promoter *POmpc*, which could be a strategy to develop tumor environment specific therapeutics (116).

**Table 1:** A combined list of bacteria currently under different phases of Clinical trials.

Intervention	Study Title	Phase	Condition	NCT Number
Bacterial cellulose-monolaurin hydrogel	Bacterial Cellulose-monolaurin Hydrogel for Preventing Therapy-induced High-grade Acute Dermatitis Among Filipinos with Breast Adenocarcinoma: A Pilot Randomized Controlled Trial	Phase 2	Acute Radiation Dermatitis	NCT05079763
*Clostridium Novyi-NT*	Pembrolizumab With Intratumoral Injection of *Clostridium Novyi-NT*	Phase 1b	Malignant Neoplasm of Breast, digestive Organs, eye, brain and other Parts of Central Nervous System	NCT03435952
*Salmonella* VNP20009	VNP20009 in Treating Patients with Advanced or Metastatic Solid Tumors That Have Not Responded to Previous Therapy	Phase 1	Unspecified Adult Solid Tumor	NCT00004216
*Salmonella typhimurium*	IL-2 Expressing, Attenuated Salmonella Typhimurium in Unresectable Hepatic Spread	Phase 1	Liver Cancer	NCT01099631
*Salmonella* VNP20009	Treatment of Patients with Cancer with Genetically Modified *Salmonella Typhimurium* Bacteria	Phase 1	Neoplasm Metastasis	NCT00004988
*Clostridium butyricum* CBM 588 Probiotic Strain	CBM588 in Improving Clinical Outcomes in Patients Who Have Undergone Donor Hematopoietic Stem Cell Transplant	Phase 1	Hematopoietic and Lymphoid Cell Neoplasm	NCT03922035
*Clostridium novyi*-NT spores	Safety Study of Intratumoral Injection of Clostridium Novyi-NT Spores to Treat Patients with Solid Tumors That Have Not Responded to Standard Therapies	Phase 1	Solid Tumor Malignancies	NCT01924689
Drug: SYNB1891 Drug: Atezolizumab	Safety and Tolerability of SYNB1891 Injection Alone or in Combination with Atezolizumab in Adult Subjects	Phase 1	Metastatic Solid Neoplasm, Lymphoma	NCT04167137
*Listeria monocytogenes*	Phase 3 Study of ADXS11-001 Administered Following Chemoradiation as Adjuvant Treatment for High Risk Locally Advanced Cervical Cancer: AIM2CERV	Phase 3	Cervical cancer	NCT02853604

Host microbiome has recently been studied as an important contributor to tumorigenesis and tumor progression (62, 117) and can even negatively affect chemotherapy (118–121). The mechanism by which bacterium like *Bacteriodes fragilis, Escherichia coli, Campylobacter jejuni*, *Fusobacterium nucleatum, Salmonella typhi, Helicobacter pylori* contributes to tumorigenesis and progression has been reported and attributed to factors like (a) Secretion of proteins, secondary metabolites like toxins and reactive oxygen species which contribute to DNA damage and genomic instability and accelerates tumorigenesis (122–129), (b) altering host immune regulation pathways and upregulates pro- oncogene like *cyclin D* and *c- Myc* (130, 131), (c) promoting exosome secretion enriched in miR- 1246/92b-3p/27a-3p which promotes tumor metastasis (132, 133). Understanding the genotypic makeup of these bacterium which enables them to localize within the tumor can help us further fine tune targeted bacterial therapy in the future.

Apart from genetically modified bacteria, functionalized bacteria has been designed to achieve accurate delivery and controlled release of drugs and maintaining excellent biocompatibility (134). Although bacteria mediated regression of tumors has been established in experimental models and have been demonstrated to be well tolerated by the host, there are unanswered questions related to the technique. Firstly, like traditional approaches, most of the administered bacteria can be unavoidably eliminated by reticuloendothelial system before arrival at the site of tumor. Secondly, bacterial propagation would not lead to proliferation of therapeutic material resulting in its dilution. Lastly, bacteria induced systemic infection is another potential hazard and carries a significant risk. Bacterial mediated cancer therapy is in its infancy, but holds immense potential to change the current strategies of cancer treatment. As compared to treatment methods like Fecal microbial transplant (FMT), genetically modified bacteria holds certain advantage. FMT involves transfer of fecal microbiota from healthy individuals has showed encouraging results in patients with clostridiodes difficile infection, inflammatory bowel disease, irritable bowel syndrome, multiple sclerosis, hepatic encephalopathy, Parkinson’s disease and diabetic neuropathy (135–141). The efficacy of FMT as an anti- cancer therapy still under studies in conjugation with immune checkpoint blockade inhibitors like anti- PD1/PD-L1 or anti- CTLA4 antibodies (142–145) and are in different phases of clinical trials (NCT03353402, NCT03341143, NCT03772899, NCT03819296, NCT04577729, NCT04116775, NCT04758507, NCT04951583, 04988841, NCT05286294, NCT05279677, NCT0438619, NCT05008861, NCT04521075, NCT03819296, NCT05251389). However, presence of drug- resistant microbes in the microbiota of otherwise healthy patient have been reported to cause clinically life- threatening complications undergoing cancer treatment (146). Furthermore, microbiome varies from individual to individual which can effect the efficacy of the treatment (147, 148). Bacteria based cancer therapy on the other hand involves genetic engineering which alter the genetic makeup of the bacteria to nullify it’s virulence and safe for clinical applications. Further combinatorial therapy involving bacteria and conventional therapy involving radio and chemotherapy have also been implemented by research groups in animal models and human patients which have yielded promising results (**Table 2**).

**Table 2:** A list of bacterial therapy in combination with traditional radiation or chemotherapy.

S.No	Bacterial strain and route of injection	Combinatorial therapy	Outcome
1	*Salmonella typhimurium* SHJ2037 (I.V)	Radiotherapy with 21Gy in mice bearing CT26 colon cancer	Tumor regression (149)
2	*Salmonella* YS146 and YS166 (I.P or I.V)	X- ray irradiation with 5- 15 Gy in mice bearing B16F10 or Cloudman S91 melanomas	Tumor suppression, prolonged survival and supra- additive anti- tumor efficacy (150)
3	*E. coli* K12- expressing *ClyA* gene (S.C)	Radiation with 21Gy in mice bearing CT26 colon cancer	Suppression of metastatic tumor growth in lung and prolonged survival (151)
4	*Clostridium oncolyticum* M55 (I.V)	Local tumor hyperthermia by radio frequency in mice bearing Ehrlich solid carcinoma, Harding- Passey- melanoma, fibrosarcoma, neck tumor or local tumor hyperthermia with local X- ray irradiation in mice bearing Harding- Passey melanoma	Oncolysis of tumors and improvement in survival rate (152–154)
	*Clostridium novyi*- NT (I.V)	Irradiation with 0.1 Gy, systemic radioimmunotherapy with I- 131 conjugated mAb and brachytherapy using plaques loaded with I- 125 seeds in nude mice bearing HCT116 tumors, HuCC-T1 xenografts, LS174T xenograft	Shrinkage of tumors and enhanced survival as compared to bacterial therapy alone (155)
5	*Salmonella TAPET- CD* expressing *cytosine deaminase* gene of *E. coli* (I.T)	Combinatorial chemotherapy with 5- fluorocytosine (5- FC) in refractory cancer patients	Bacterial colonization within tumor sites and enhanced production of 5- fluorouracil (5- FU) (156)
6	*Salmonella typhimurium* VNP20009 (I.P)	In combination with cyclophosphamide in B16F10 murine melanoma model	Decrease in tumor microvessel density, serum vascular endothelial growth factor (VEGF) inhibited tumor growth and enhanced survival. Enhanced bacterial localization within tumor (157).

## Conclusion

Owing to properties like hypoxia targeting, motility, immunogenicity, and ability to deliver oncolytic genes or drugs, bacteria mediated cancer immunotherapy can be regarded as a promising approach. Several genetically engineered bacteria are already in the clinical trials and their outcome will provide a much-awaited proof of concept for the utility of this approach as anticancer therapeutics. These milestones will enable researchers to establish safety dosage, administration, feasibility of combining bacterial immunotherapy with conventional therapy, and upstream processing. Optimizing these parameters will open up a new avenue of cancer therapeutics and address the unmet needs of patients.

## Author contributions

HG: Conceptualization, Funding acquisition, Methodology, Software, Writing – review & editing. SS: Writing – original draft, Reviewing. HS: Writing – original draft, Writing – review & editing.
